# A Comprehensive Review of the Approach to Ultrasonography in Neonatal Shock: The Partnership Between Targeted Neonatal Echocardiography and Point-of-Care Ultrasound

**DOI:** 10.3390/children13091268

**Published:** 2026-09-18

**Authors:** Nina Nouraeyan, Kimberly Wong, Ashraf Kharrat, María V. Fraga, Gabriel Altit

**Affiliations:** 1 Division of Neonatal Perinatal Medicine, Jewish General Hospital, McGill University, Montreal, QC H3T 1E2, Canada; nina.nouraeyan@mcgill.ca; 2 Division of Neonatal Perinatal Medicine, Montreal Children’s Hospital, McGill University, Montreal, QC H4A 3H9, Canada; kimberly.wong4@mcgill.ca; 3 Department of Pediatrics, Mount Sinai Hospital, University of Toronto, Toronto, ON M5G 1X5, Canada; ashraf.kharrat@sinaihealth.ca; 4 Department of Pediatrics, Children’s Hospital of Philadelphia, Perelman School of Medicine, University of Pennsylvania, Philadelphia, PA 19104, USA; fragam@chop.edu

**Keywords:** point-of-care ultrasound, targeted neonatal echocardiography, neonatal shock

## Abstract

**Highlights:**

**What are the main findings?**

• Bedside ultrasound can provide real-time insight into the complex and evolving physiology of neonatal shock. Point-of-care ultrasound (POCUS) and targeted neonatal echocardiography (TNE) offer different but complementary information that can help clinicians understand the cause of deterioration and guide individualized management.
• POCUS provides rapid, focused assessment of cardiac and non-cardiac causes of deterioration, while TNE provides a more detailed assessment of cardiovascular physiology and hemodynamic phenotype.
• Serial ultrasound assessment can help clinicians understand evolving shock physiology and evaluate the response to interventions, including fluid, vasoactive, and ventilatory therapies.

**What are the implications of the main findings?**

• POCUS and TNE should not be viewed as competing modalities but as complementary tools that answer different clinical questions in the assessment of critically ill neonates.
• Using an integrated approach of POCUS and TNE in the evaluation of ill neonates at bedside may facilitate identification of reversible causes of deterioration and real-time guidance of management.

**Abstract:**

The neonatal cardiovascular system is dynamic and influenced by multiple factors, which makes neonatal shock a challenging early clinical diagnosis. Real-time monitoring has been limited to indirect measurements of hemodynamic well-being, such as heart rate, blood pressure, capillary refill time, and biochemical markers of adequate oxygen delivery and oxygen consumption. Bedside diagnostic ultrasound to guide decision-making has become increasingly integrated into modern neonatal care. It allows clinicians to enhance physiologic monitoring, differentiate among diverse cardiovascular phenotypes, including time-critical conditions such as tamponade, that may present with similar clinical signs, and interpret complex, evolving physiology in real time. By providing this additional layer of physiologic insight, it may enable more precise, individualized management, with potential implications for fluid administration, vasoactive support, and ventilatory strategies. Among neonatologists who incorporate bedside imaging into clinical practice, some rely on focused point-of-care ultrasound (POCUS) skills, while others have formal training in targeted neonatal echocardiography (TNE). This review outlines the distinct but complementary roles of POCUS and TNE, highlighting the specific diagnostic questions each can address, as well as their respective limitations in the evaluation of neonatal shock. Broadly, POCUS is designed to rapidly identify causes of clinical deterioration through focused lung, cardiac, abdominal, and cranial assessments. In contrast, TNE provides a more comprehensive and longitudinal evaluation of cardiovascular function guiding management over time. Differences in training pathways may create a perceived dichotomy between POCUS and TNE; however, these modalities are inherently complementary and should be integrated in the care of critically ill infants. Ultimately, the neonate must be assessed holistically, with ultrasound serving to augment—rather than replace—clinical judgment within the broader clinical context. A clear understanding of the interdependent roles of POCUS and TNE is essential to enable timely, meaningful, and integrated use of bedside ultrasound in the neonatal intensive care unit.

## 1. Introduction

Neonatal physiology evolves rapidly and continuously during the transition to extrauterine life, especially in the context of medical or surgical complexity. These adaptations affect every organ system as the newborn adapts to the postnatal environment and shifts from fetal to neonatal circulation. Within the cardiovascular system, these changes are highly dynamic, influenced by multiple factors, and substantially alter hemodynamics, including preload, afterload, myocardial performance, and shunt physiology. Under conditions of physiological stress, dysfunction in other organ systems may further contribute to maladaptation, thereby impacting overall circulatory stability. In both term and preterm infants, reliable clinical signs to accurately assess cardiovascular physiology during illness are limited, making the diagnosis of shock particularly challenging and often delayed until a stage when tissue ischemia is already established [1,2]. In its simplest form, shock is defined as a state of circulatory insufficiency in which oxygen delivery is inadequate to meet tissue metabolic demands. However, commonly used non-invasive hemodynamic measures—such as continuous heart rate, blood pressure, and capillary refill time—are indirect, subjective, and imprecise measures of tissue perfusion, especially during the critical period of postnatal adaptation [3]. Consequently, neonatologists often face difficulty in accurately determining the hemodynamic status of critically ill infants without deeper insight into the interplay between systemic and pulmonary vascular resistance, intra- and extracardiac shunt physiology, volume status, myocardial function, intra- or extracardiac obstructive compromise, and peripheral perfusion [3]. In response to these challenges, diagnostic ultrasound to aid in decision-making has evolved rapidly over recent decades, complementing bedside clinical assessment in high-risk newborns with pathological processes that lead to evolving or established shock physiology. In neonatology, its application is broadly categorized into two complementary domains: hemodynamic assessment through targeted neonatal echocardiography (TNE)/neonatologist-performed echocardiography [4,5] and broader bedside evaluation through point-of-care ultrasound (POCUS) [6,7,8,9,10,11]. Recent expert consensus and editorial work have emphasized the importance of clearly delineating the respective roles, scope, and limitations of POCUS and TNE while recognizing their complementary nature in clinical practice [12]. The goal of this review is to explore this complementarity in the context of neonatal shock and evolving cardiovascular collapse, highlighting how each modality can be integrated to support timely, physiology-driven decision-making.

## 2. Literature Search Strategy

A narrative literature search was conducted in PubMed using combinations of the terms “point-of-care ultrasound,” “POCUS,” “targeted neonatal echocardiography,” “TNE,” “neonate,” “neonatal,” and “shock.” Retrieved publications were reviewed for relevance to the assessment and management of neonatal shock. As this was a narrative rather than a systematic review, formal inclusion and exclusion criteria were not applied. Literature was selected based on relevance to the clinical questions addressed in this review, with emphasis on key clinical studies, consensus statements, guidelines, and publications considered pertinent based on the authors’ expertise in neonatal POCUS and TNE.

## 3. Complementary Roles of TNE and POCUS in Neonatal Shock Assessment

POCUS is a question-driven modality designed to address focused, clinically relevant problems in real time. Its scope is broad, encompassing cranial, pulmonary, gastrointestinal, bladder, and simplified cardiac imaging, as well as procedural guidance [9,11]. POCUS allows clinicians to rapidly obtain physiological information that complements bedside assessment and supports individualized care [6]. While it is not intended to provide a comprehensive functional hemodynamic or structural cardiologic evaluation, POCUS can play an important adjunctive role in the rapid assessment of infants in shock, helping to guide immediate management decisions.

TNE is performed by neonatologists who have undergone advanced, dedicated training in cardiovascular physiology and echocardiographic functional assessments. It represents a comprehensive bedside application of ultrasound to evaluate cardiac systolic and diastolic function, preload, systemic and pulmonary blood flow, intra- and extracardiac shunts, and pericardial effusion. TNE enables a detailed understanding of cardiovascular physiology and supports targeted, timely, and serial assessments of therapeutic interventions. Over the past decade, there have been significant efforts toward standardization of TNE training, resulting in structured programs and accreditation pathways across North America [4]. Given the depth of expertise required, TNE remains a specialized skill that requires additional dedicated training.

Similarly, efforts toward standardized training, credentialing, and quality assurance in neonatal POCUS have expanded in recent years [8], with emerging guidelines supporting its application in the evaluation and management of shock [10].

Importantly, neither cardiac POCUS nor TNE replaces comprehensive echocardiography performed by pediatric cardiology. Cardiac POCUS addresses focused bedside questions, and TNE provides detailed functional and hemodynamic assessment, whereas pediatric cardiology echocardiography provides comprehensive structural and diagnostic cardiac evaluation [4,12].

Together, POCUS and TNE offer complementary and synergistic insights into neonatal cardiovascular compromise, particularly in critically ill infants presenting with shock, while remaining distinct from and not replacing comprehensive echocardiographic assessment by pediatric cardiology.

## 4. Types of Neonatal Shock

### 4.1. Definition of Shock

Shock is defined as circulatory failure where oxygen demand and supply are mismatched [13]. This inability to meet the metabolic demands of the body leads to rising lactate and end-organ damage.

### 4.2. Distributive and Septic Shock

Septic shock has traditionally been categorized into “warm” and “cold” phenotypes.

Warm shock, or distributive shock, is characterized by decreased systemic vascular resistance (SVR), leading to warm extremities, brisk capillary refill, edema, bounding pulses, and low diastolic blood pressure [13]. These infants may exhibit hyperdynamic cardiac contractility, as the left ventricle compensates for the underfilling caused by the reduction in SVR [14].

In contrast, cold shock reflects a primarily increased SVR state and presents with cool extremities, delayed capillary refill, weak pulses, and eventual hypotension [14]. These infants may develop left ventricular or biventricular dysfunction, often compounded by reactive pulmonary hypertension.

However, this binary classification of “warm” vs. “cold” oversimplifies the complex and evolving hemodynamic profiles observed in neonates and may lead to suboptimal therapeutic decisions regarding inotropes, vasopressors, inodilators, corticosteroids, or fluid management [14].

### 4.3. Hypovolemic Shock

Volume status is another key determinant of neonatal shock, either as a primary driver or as a secondary consequence of underlying pathology [13]. Hypovolemia may result from blood or fluid loss, including placental or cord-related events, fetomaternal hemorrhage, and internal or external bleeding (e.g., intracranial, pulmonary, or subgaleal hemorrhage) [15,16,17].

### 4.4. Cardiogenic Shock

Cardiogenic shock represents a critical and often rapidly progressive etiology in newborns resulting from primary right, left, or biventricular dysfunction [13]. It may arise from maladaptation during the transitional period, ischemic or inflammatory myocardial injury (including hypoxic–ischemic events), infectious myocarditis, abnormalities in coronary perfusion, or, more rarely, congenital or genetic cardiomyopathies [18,19,20,21]. Structural heart disease, whether undiagnosed or acutely decompensated, remains an important consideration, particularly in duct-dependent lesions or conditions associated with impaired systemic or pulmonary blood flow [19].

### 4.5. Obstructive Shock

Obstructive shock in newborns results from a mechanical impediment to cardiac filling or output, leading to reduced effective circulation despite preserved or even increased myocardial contractility [13]. In the neonatal population, this may occur in the setting of pericardial tamponade (e.g., secondary to central line malplacement, hydrops, lymphatic disorders, or after cardiac interventions), tension pneumothorax, hypertrophic cardiomyopathy, or critical structural lesions that obstruct systemic or pulmonary blood flow [22,23,24]. Rapid recognition is essential, as management requires prompt relief of the mechanical obstruction, such as pericardiocentesis, thoracentesis, heart rate modulation, prostaglandin infusion for duct-dependent lesions, or urgent surgical or catheter-based intervention, rather than conventional fluid or inotropic therapy alone [16,23].

### 4.6. Dissociative Shock

This refers to a state in which oxygen delivery may be relatively preserved, but effective tissue oxygen utilization or distribution is impaired, leading to cellular hypoxia [13]. This can occur in conditions such as severe anemia (e.g., fetomaternal hemorrhage) or methemoglobinemia, where the oxygen-carrying capacity or hemoglobin function is compromised despite adequate cardiac output [25].

**Figure 1:**
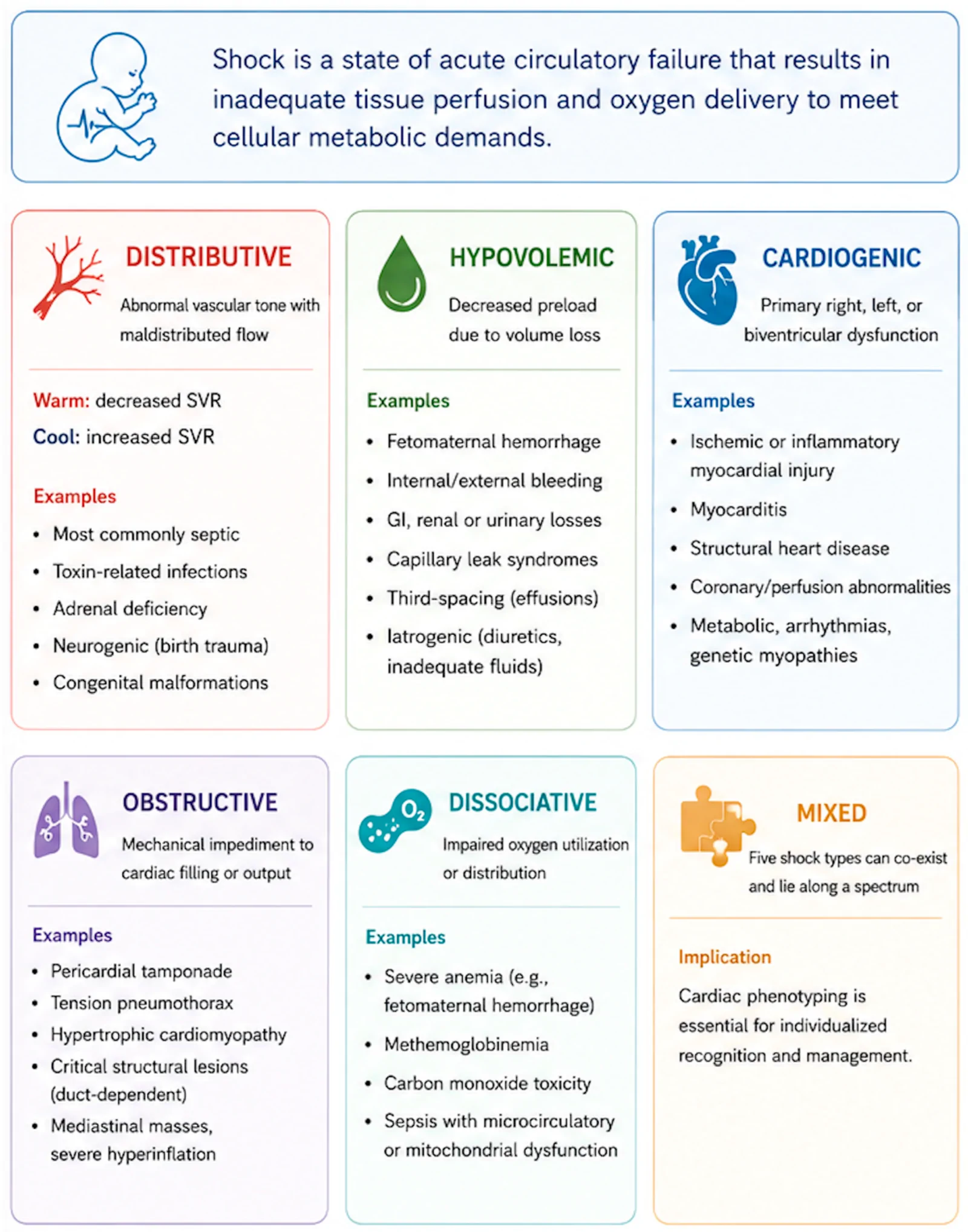
Review of types of neonatal shock.

Importantly, the management of these shock states (Figure 1) differs, and failure to accurately distinguish between these entities may result in inappropriate therapies, including excessive fluid administration or suboptimal vasoactive support. In this context, bedside ultrasound may provide valuable complementary information. This review aims to explore how both POCUS and TNE contribute to the assessment of neonatal shock, emphasizing their distinct yet complementary roles in refining the diagnostic approach and guiding physiology-based management in this vulnerable population.

## 5. POCUS in Neonatal Shock: A Structured, Rapid Diagnostic Adjunct

POCUS provides rapid, bedside, problem-oriented information to identify reversible cardiac and non-cardiac contributors to clinical deterioration. It enables clinicians to quickly detect alternative or coexisting pathologies that may significantly alter management and support a more individualized approach to care. Its value may be particularly relevant in cases of shock refractory to initial resuscitation, where it can help uncover the underlying cause of deterioration.

Structured, single-probe, stepwise protocols, such as the Sonographic Assessment of Life-Threatening Emergencies—Revised (SAFE-R), have been developed to assess the acutely decompensating (i.e., rapid cardiovascular collapse) neonate [26]. These algorithms prioritize the rapid exclusion of immediately reversible, life-threatening conditions through targeted imaging of key anatomical regions. For example, the first step in the SAFE-R approach is the exclusion of a significant pericardial effusion causing possible tamponade physiology, using a subcostal cardiac view [26]. Although rare in neonates, tamponade represents a critical and treatable cause of shock, particularly in the context of central line malposition [16]. Importantly, this focused assessment for the detection of significant effusions can be reliably performed by operators with basic ultrasound training, enabling broad applicability across units providing advanced neonatal care [4].

Given that a substantial proportion of neonatal cardiovascular compromise is secondary to respiratory pathology, the next steps of the SAFE-R algorithm focus on lung/thoracic assessment [26]. Lung POCUS is a sensitive and accessible tool that can rapidly differentiate causes of respiratory deterioration [27,28,29]. In observational studies, it has demonstrated high diagnostic accuracy for pneumothorax, comparing favorably with chest radiography and transillumination, allowing prompt identification of the need for decompression [30,31]. Additionally, thoracic ultrasound can detect significant pleural effusions, which may be difficult to appreciate on supine radiographs [32,33]. In neonates, these may arise from congenital conditions such as chylothorax [34], postoperative complications [35], or central line extravasation [36], all of which can precipitate acute decompensation [37]. The speed and diagnostic clarity provided by lung POCUS make it a cornerstone of the SAFE-R algorithm and an important step in the evaluation of an infant with undifferentiated shock.

The protocol then extends to the assessment of systemic blood flow by evaluating the abdominal aorta [26]. In the transitional circulation, critical left-sided obstructive lesions, such as coarctation of the aorta, may present with sudden cardiovascular collapse [38]. The absence of a pulsatile abdominal aorta (which may correlate with absent or diminished femoral pulses and with pre- and post-ductal blood pressure differences) raises concern for duct-dependent systemic circulation and may warrant urgent initiation of prostaglandin therapy while awaiting confirmatory evaluation by pediatric cardiology [26,39]. While this assessment does not replace formal echocardiography for congenital heart disease, it provides a rapid screening tool to guide early management decisions [4].

Further evaluation includes assessment for intra-abdominal free fluid [40]. Hemoperitoneum or significant intra-abdominal fluid accumulation, whether due to birth trauma, hydrops, lymphatic disorder, hepatic injury, bladder rupture, or vascular line extravasation, can result in significant intravascular volume loss and contribute to hypovolemic shock [26].

Cranial ultrasound also plays an important role, particularly in preterm infants or those with perinatal compromise. Acute intracranial hemorrhage, especially from the germinal matrix, may lead to significant blood loss, anemia, and acidosis, thereby contributing to hemodynamic instability [41]. In addition, evolving complications such as post-hemorrhagic hydrocephalus may present with subtle clinical signs, including lethargy, or with recurrent and progressively severe apnea and bradycardia episodes that may be interpreted as cardiopulmonary instability; these conditions can be rapidly identified through bedside cranial ultrasound [26].

Although not exhaustive, this structured approach illustrates how POCUS can identify key, potentially reversible causes of shock that may not be apparent through cardiovascular assessment alone. For example, TNE may identify low cardiac output but would not detect pleural or abdominal extravasation of fluids from mispositioned central lines unless the practitioner is also trained in other POCUS applications. Similarly, ensuring appropriate positioning of endotracheal tubes [42] (e.g., bilateral lung sliding) and vascular access devices [43,44] is critical in the evaluation of a deteriorating neonate and can be rapidly assessed using ultrasound.

Beyond its diagnostic value, POCUS is relatively accessible and can be incorporated into neonatal practice with appropriate training [11]. Recently published guidelines support its integration into educational curricula and clinical workflows [7,8]. While barriers to implementation remain, there is increasing recognition of its importance in enhancing bedside decision-making. Neonatologists with advanced TNE training are well positioned to support the integration of POCUS into clinical practice, leveraging their expertise in ultrasound physics and image acquisition. When integrated thoughtfully, POCUS and TNE may provide a comprehensive, physiology-driven framework for the evaluation and management of neonatal shock, which may support more precise and individualized care.

## 6. TNE: Cardiovascular Phenotyping for the Physiology-Driven Management of Neonatal Shock

### 6.1. Current Landscape of TNE

TNE emerged in response to the recognized limitations of traditional clinical markers, such as heart rate, blood pressure, capillary refill time, and urine output, in accurately reflecting circulatory status in term and preterm infants [3,4]. While these parameters may signal early deterioration, they lack the specificity and the positive predictive value required to guide individualized management. TNE helps to address some of these gaps by offering real-time, physiology-based insights to inform targeted interventions, including fluid therapy, cardiopulmonary interactions, and vasoactive support. The field has expanded, with increasing research, formalized training programs, and a growing presence of TNE-trained neonatologists globally [4].

Dedicated fellowship programs and evolving guidelines now define the role of the neonatal hemodynamics consultant within the NICU [4]. Training is typically one year and includes hands-on bedside experience and structured exposure to pediatric echocardiography laboratories alongside cardiologists and cardiac sonographers, ultimately intended to prepare a trainee for consultative clinical practice. In Canada, neonatal hemodynamics has been recognized as an Area of Focused Competence (AFC) by the Royal College of Physicians and Surgeons of Canada, reflecting a nationally accredited, advanced subspecialty with defined standards aimed at improving quality of care and patient safety [45].

TNE is particularly valuable in the comprehensive phenotyping of neonatal shock, addressing key hemodynamic domains that may shift during illness: volume status, biventricular function, shunt physiology, cardiac output estimation, inter-ventricular interactions, and flow distribution, as well as monitoring of the response to individualized therapeutic interventions [5]. A central challenge in neonatal shock is distinguishing between underlying etiologies and their respective contributions in multifactorial processes, such as distributive, hypovolemic, obstructive, and/or cardiogenic shock, which requires integrated assessment of cardiac output, vascular resistance, and preload conditions.

### 6.2. TNE in Neonatal Shock

TNE allows clinicians to understand the pathophysiology of hemodynamic compromise with the goal of tailoring and monitoring interventions. This field is currently evolving, and normative values for many parameters are still being established and validated [46,47]. When used serially, TNE can follow the impact of an intervention and help visualize the evolution of a patient in ways that clinical signs alone may not allow.

In neonatal sepsis, fluid resuscitation is commonly a first-line intervention. Assessment of volume status remains complex in neonates both clinically and with TNE. Unlike in older populations, inferior vena cava (IVC) evaluation has not been validated across gestational age, necessitating alternative or complementary approaches [48,49]. Transitional physiology in the early neonatal period, when the right ventricle has not yet undergone postnatal remodeling toward improved compliance, combined with interatrial shunting, tricuspid regurgitation, and the effects of positive pressure ventilation, can significantly influence right ventricular end-diastolic pressure and right atrial filling pressures. As a result, IVC size may not reliably reflect true intravascular volume status. The IVC distensibility index (IVCDI) is used in mechanically ventilated patients, whereas the IVC collapsibility index (IVCCI) is used in spontaneously breathing neonates; both are derived from the maximal and minimal IVC diameters measured in the subcostal view [50]. In ventilated neonates, particularly with high or sustained mean airway pressure, the IVCDI may not be reliable [51]. In the absence of validated thresholds, the potential value of IVC assessment by TNE lies mainly in serial evaluation, particularly in neonates who may be sensitive to small changes in volume status.

To evaluate cardiac output, various calculations can be made, each with its own validation and limitations. In the absence of significant shunts, left ventricular output approximates systemic blood flow, whereas right ventricular output reflects venous return. When evaluating left ventricular function, it is important to recall that the movement of the ventricle is along three planes: circumferential, longitudinal, and radial, and the inflow and outflow tracts differ in their myocardial fiber architecture [52]. Therefore, dysfunction in one plane may not reflect dysfunction in another, making estimations of cardiac output dependent on where measurements are taken.

Left ventricular function can be assessed using surrogates of stroke volume, such as left ventricular outflow tract velocity–time integral (LVOT VTI) in combination with LVOT diameter, both measured just proximal to the aortic valve in the 5-chamber view and long axis view, respectively [53,54]. Normal values of LVOT VTI and cardiac output change during the transition from fetal to extrauterine life and with postnatal age and should be interpreted accordingly [46,47].

Ejection fraction can be assessed using four-chamber views in both 2D and 3D, and speckle tracking can assess myocardial strain and ventricular dysfunction [55]. Shortening fraction, which is derived from the left ventricular end-diastolic and end-systolic diameters, can be measured in the parasternal short- or long-axis view and is a marker of ventricular systolic function [56]. The value of these methods lies in determining whether left ventricular function is above or below the normal range and, more importantly, whether the ventricle is responding to inotropic support or volume expansion as expected.

The right ventricular outflow tract velocity–time integral (RVOT VTI) and the tricuspid annular plane systolic excursion (TAPSE) also allow identification of right-sided strain and possible pulmonary hypertension leading to right-sided failure [54,57]. Furthermore, the tricuspid regurgitation (TR) jet velocity can be used to estimate right ventricular systolic pressure and help identify systemic or suprasystemic pulmonary hypertension.

In low SVR states, such as septic shock, with a predominant distributive picture, TNE can help delineate the hemodynamic phenotype [58]. In the setting of low SVR, affected infants may initially exhibit a hyperdynamic circulatory profile. Due to venodilation and third spacing, the ventricles may appear subjectively underfilled, with increased apposition of the myocardial walls, while conventional echocardiographic markers of systolic performance, such as shortening fraction and ejection fraction, may appear augmented.

As the left ventricle (LV) is contracting against a markedly reduced afterload, this apparent preservation or enhancement of systolic function may be misleading. Tachycardia commonly develops as a compensatory response to sensed falling perfusion pressure or metabolic demand and represents the principal mechanism by which the newborn increases cardiac output; through the force–frequency relationship, a higher heart rate may transiently augment contractility and support left ventricular output (although this force-frequency relationship may be blunted in newborns) [59,60,61]. Recognition of this phenotype may support a physiology-based strategy of increasing SVR with vasopressor therapy to restore perfusion pressure. Particularly in the context of ongoing inflammation and continued effects of the underlying infectious insult, this approach requires careful reassessments. Serial TNE may therefore be valuable in infants who remain unstable despite initiation or escalation of treatment, as restoration of SVR may unmask evolving LV dysfunction.

### 6.3. Implications of Shunt Physiology in Inflammatory States

The presence of extra-cardiac shunts, most commonly a patent ductus arteriosus (PDA), may further modify hemodynamics and influence therapeutic decision-making. Inflammatory states may render the ductus more unrestrictive, and when the relationship between SVR and pulmonary vascular resistance (PVR) favors ductal steal in the context of shock, this may contribute to ongoing diastolic hypotension and impaired systemic perfusion. Therapeutic strategies aimed at restoring a more favorable SVR–PVR balance may therefore be important. This includes avoiding interventions that further reduce PVR when such a reduction would worsen systemic steal, such as hypocapnia, excessive oxygen exposure beyond intended saturation targets, or agents that may increase pulmonary blood flow at the expense of systemic perfusion (e.g., pulmonary vasodilators) [62,63]. In some infants, distributive shock may coexist with an abnormal elevation in PVR relative to SVR, producing a superimposed phenotype of acute pulmonary hypertension [64]. In this setting, particularly when the ductus is unrestrictive, the low-SVR state may amplify right-to-left ductal shunting. Ductal flow is determined by the intrinsic resistance of the ductus itself, which depends on its diameter and length, by blood viscosity, and by the relative relationship between PVR and SVR. Increasing SVR may reduce the magnitude of right-to-left ductal shunting and thereby help preserve pulmonary blood flow.

### 6.4. Selection of Vasoactive Therapy in Low Cardiac Output States

As outlined, vasoactive therapy should be individualized according to the underlying cardiovascular physiology. Although dopamine has historically been used as a first-line agent because of its blood pressure–augmenting effects [16,60], its tendency to increase PVR has raised concern regarding potential harm in infants with already elevated PVR [64]. Conversely, that same pharmacologic effect may be advantageous in selected infants with a large left-to-right PDA and systemic steal physiology. Increasingly, a physiology-driven, TNE-guided approach to vasoactive selection has been proposed as an alternative to a standardized dopamine-centric strategy, although comparative outcome data remain limited. The choice of vasoactive support should therefore consider SVR, PVR, shunt physiology, and ventricular function, with serial evaluations to guide an individualized approach.

### 6.5. Pulmonary Hypertension Assessment in Neonatal Shock

Pulmonary hypertension (PH) represents an additional and often highly dynamic component of neonatal shock. It may arise secondary to inflammation, hypoxemia, or acidosis [64]. POCUS can provide rapid qualitative clues, such as the presence of ventricular dysfunction, tricuspid regurgitation, or systolic septal flattening, whereas TNE allows a more refined assessment, including estimation of right ventricular (RV) systolic pressure from tricuspid regurgitation jet velocity and evaluation of ventricular–vascular interactions/coupling. Conceptually, pulmonary arterial pressure reflects the interaction among pulmonary blood flow, PVR, and distal drainage pressure (i.e., pulmonary capillary wedge pressure). In neonates with cardiovascular decompensation and acute PH, abnormalities in any one or several of these components often coexist and rarely occur in isolation [65,66]. The presence of a widely patent ductus further complicates interpretation. When the PDA is unrestrictive, it effectively equalizes pressure between the pulmonary artery and the aorta, such that the relative relationship between PVR and SVR determines the magnitude and direction of shunting. This may fluctuate not only from beat to beat but also across the cardiac cycle, for example, with right-to-left shunting in systole and left-to-right shunting in diastole.

### 6.6. Implications of Ductal Physiology in Pulmonary Hypertension

Ductal caliber is itself dynamic, often larger in systole because of distending pressure, and may also vary with constriction or reopening under the influence of inflammatory mediators [67,68]. As the duct progressively constricts, pressure can no longer equilibrate freely across the two circulations. Under those conditions, vascular resistance becomes a stronger determinant of compartmental pressure, such that elevated PVR may drive a rise in pulmonary arterial pressure, whereas low SVR may contribute to a fall in aortic pressure. Excessive pulmonary pressure loading may overwhelm the right ventricle, leading to RV failure [64]. As RV end-diastolic pressure rises, right atrial filling pressure also increases, and depending on the size of the interatrial communication, deoxygenated blood may increasingly shunt right-to-left across the atrial septum, thereby worsening systemic hypoxemia. In contrast, when pre- or post-tricuspid shunts are small or absent, there may be limited capacity for decompression of the right-sided circulation. In such cases, severe RV failure may precipitate catastrophic low cardiac output, poor systemic perfusion, and refractory acidosis because pulmonary blood flow and, consequently, pulmonary venous return to the LV acutely fall [64]. Similarly, progressive LV dysfunction in cardiogenic shock may lead to increased LV end-diastolic pressure, impaired coronary perfusion, particularly when diastolic aortic pressure is low, and transmission of elevated filling pressures to the left atrium, thereby generating a post-capillary component of PH. Left atrial pressure, however, may remain partially decompressed if the interatrial shunt is sufficiently large to offload the left atrium. TNE provides a comprehensive framework to assess these dynamic elements, including estimation of RV and pulmonary arterial pressures, evaluation of RV performance, and integration of shunt physiology [69]. Importantly, no single echocardiographic parameter should be interpreted in isolation. Rather, findings must be integrated to formulate a complete assessment of the underlying cardiovascular state.

### 6.7. Visceral and Cerebral Arterial Doppler

Beyond central cardiac indices, there is growing interest in the use of arterial Doppler to interrogate end-organ perfusion in the neonate. In the presence of an unrestrictive ductus arteriosus with a large left-to-right shunt, diastolic runoff into the pulmonary circulation may produce reduced, absent, or reversed end-diastolic flow with elevated resistive and pulsatility indices in the superior mesenteric, celiac, and renal arteries—a Doppler correlate of systemic diastolic steal [62,63,70]. Renal and mesenteric arterial Doppler appear more sensitive to systemic hypoperfusion than the descending aorta, in which diastolic flow reversal is a specific but relatively insensitive marker of significant steal [58,71]. Cerebral arterial flow, by contrast, is often preserved within a compensatory range by autoregulation, and the cerebral resistive index correlates only weakly with blood pressure, limiting its value as a stand-alone perfusion marker [70,72]. Elevated superior mesenteric artery indices on the first postnatal day have also been associated with subsequent necrotizing enterocolitis, though these findings are of uncertain significance once disease is established and are not validated for real-time detection of intestinal ischemia [73]. Interpretation of all of these signals is constrained by the angle of insonation, the small caliber of neonatal vessels, and organ-specific vascular resistance, and normative values remain incompletely defined across gestational age [74]. At present, therefore, visceral and cerebral arterial Doppler are best regarded as physiology-illustrating adjuncts—most informative in the context of ductal steal—rather than as validated tools to diagnose or guide the management of neonatal shock. Similarly, while the IVC collapsibility and distensibility indices offer a conceptual window on preload, no validated neonatal normative data exist, and no reliable IVC Doppler flow patterns distinguishing hypovolemic from hypervolemic states in spontaneously breathing neonates have been established [10,49,75].

### 6.8. Future of TNE

Overall, TNE enables clinicians to define the hemodynamic phenotype of shock and to guide nuanced, physiology-based management. It informs decisions regarding fluid therapy, ventilation strategies, and vasoactive support, especially in complex or evolving scenarios. Although resource- and training-intensive, TNE may be a valuable tool to support a shift beyond protocolized care toward individualized, physiology-based management in neonatal shock.

## 7. Discussion

**Figure 2:**
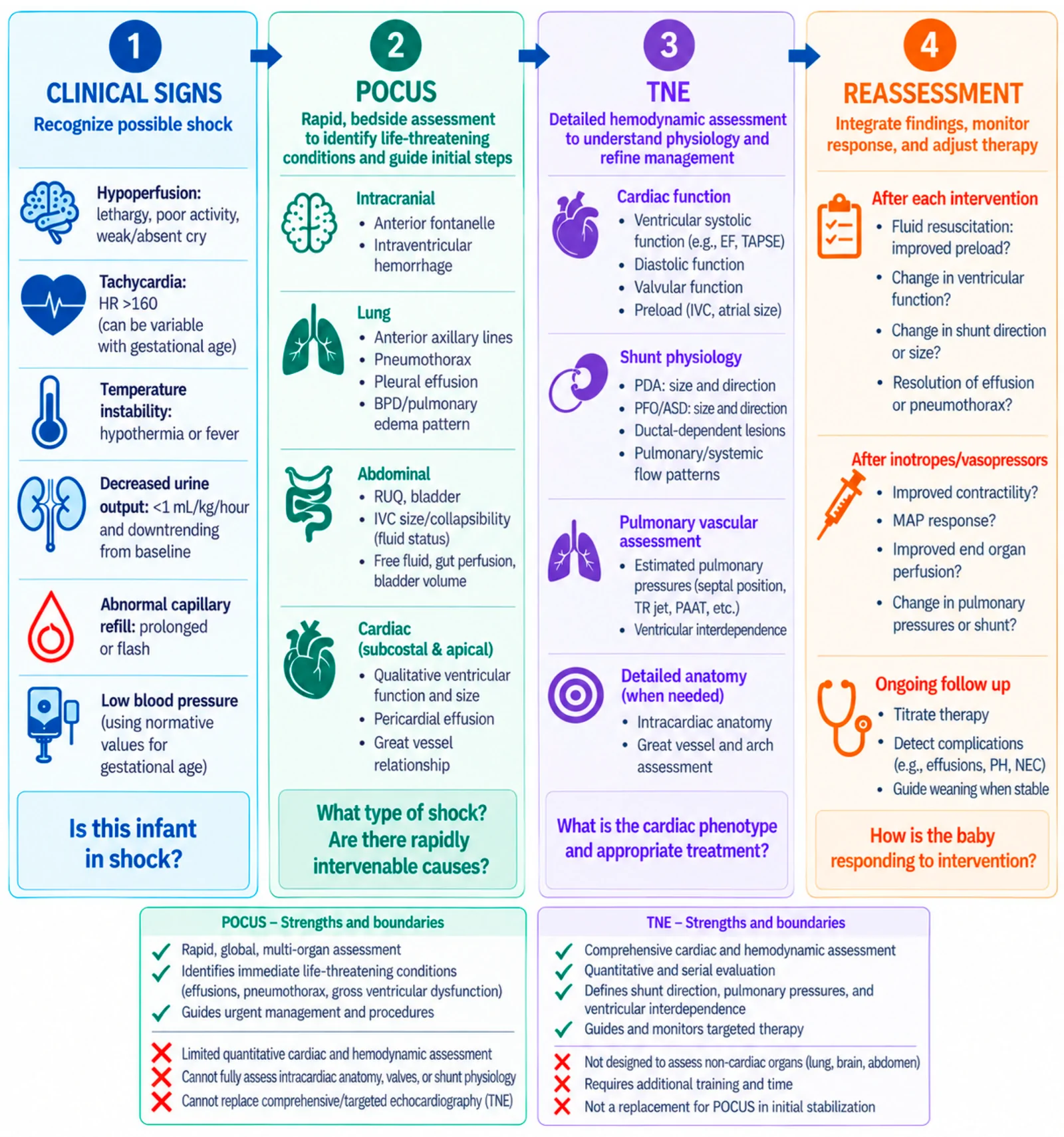
The complementary roles of point-of-care ultrasound (POCUS) and targeted neonatal echocardiography (TNE) in the assessment of a critically ill neonate.

Neonates experiencing critical physiological deterioration represent a uniquely complex population, shaped by gestational age, birth weight, intrauterine exposures, and the dynamic processes of postnatal transition. These factors interact to produce highly variable and evolving physiology, underscoring the need for individualized, physiology-based care. In this context, the complementary use of POCUS and TNE offers a promising framework for tailored clinical management, particularly during critical illness. POCUS enables rapid bedside assessment of key cardiac and extra-cardiac contributors to instability, including lung pathology, intravascular volume surrogates, global functional systolic assessment, cardiac tamponade, and abdominal conditions that may influence venous return or systemic perfusion. It is particularly valuable for identifying reversible causes of decompensation and guiding immediate interventions. In contrast, TNE provides a more detailed and structured hemodynamic evaluation, extending beyond single time-point diagnostics to allow longitudinal assessment of cardiovascular function. Through serial examinations, TNE facilitates real-time monitoring of ventricular performance, preload and afterload conditions, systemic and pulmonary blood flow, and shunt physiology, particularly in response to therapeutic interventions such as fluid administration or inotrope titration. This integrated approach is especially relevant in the management of neonatal shock, where physiologic heterogeneity demands precise characterization to guide targeted therapy. Rather than functioning as separate or competing modalities, POCUS and TNE should be viewed as complementary and interwoven tools that together provide a comprehensive physiologic assessment (Figure 2). POCUS training is increasingly being incorporated into neonatal training programs in North America and warrants continued expansion and support within neonatal intensive care units (NICUs) [7,8]. TNE, while more established, remains time-intensive and often limited to a smaller group of trained specialists. However, the evolving training landscape suggests that future neonatologists will increasingly be proficient in POCUS, with a subset pursuing advanced TNE expertise, thereby bridging both modalities and enhancing clinical and research capabilities in the field.

It is important to acknowledge the limitations inherent to both approaches. Both POCUS and TNE are inherently operator-dependent, with diagnostic accuracy influenced by image acquisition, interpretation, and the operator’s level of training and experience. Appropriate training, competency assessment, credentialing, and quality assurance are therefore essential, particularly when ultrasound findings are used to inform therapeutic decisions. Certain applications—such as intestinal ultrasound in necrotizing enterocolitis or the estimation of pneumothorax size—lack robust literature support for their validation. Interpretation is further complicated by the rapidly evolving cardiovascular physiology of the newborn, particularly during the transitional period. Gestational age, postnatal age, shunt physiology, respiratory support, and changing pulmonary and systemic vascular resistance may substantially influence ultrasound-derived measurements. Findings must therefore be interpreted within the infant’s developmental and clinical context rather than against isolated thresholds. In addition, validated neonatal reference values are lacking for several ultrasound-derived measurements, and available normative data may vary according to gestational age and postnatal transition. Consequently, individual measurements should not be interpreted in isolation or used as absolute therapeutic thresholds. A critical, question-driven approach—grounded in an understanding of these limitations—remains essential to avoid misinterpretation.

Although POCUS and TNE can provide important diagnostic and physiologic information and influence clinical management, evidence demonstrating that ultrasound-guided assessment improves short- or long-term neonatal outcomes remains limited. Available studies are predominantly observational, and further prospective investigation is needed to determine whether ultrasound-guided, physiology-based management translates into improved patient-important outcomes [76,77].

Looking ahead, an “ultrasound-first” approach to the acutely deteriorating neonate may represent a potential future paradigm, integrating rapid POCUS protocols such as SAFE-R with early targeted interventions, followed by comprehensive TNE assessment and ongoing hemodynamic monitoring by trained specialists. At present, however, this approach should be regarded as a concept requiring prospective evaluation rather than an established standard of care. Should future studies demonstrate benefit, existing resuscitation algorithms—whether for septic, hypovolemic, or cardiogenic shock—may need to evolve to incorporate ultrasound as a component of care. Ultimately, integrating POCUS and TNE not as isolated practices but as a cohesive, physiology-driven strategy holds the potential to advance neonatal care and, pending supporting outcome data, to improve outcomes for critically ill infants.

## Data Availability

No new data were created or analyzed in this study. Data sharing is not applicable to this article.

## Abbreviations

The following abbreviations are used in this manuscript:

POCUS	Point-of-care ultrasound
TNE	Targeted neonatal echocardiography
SVR	Systemic vascular resistance
SAFE-R	Sonographic Assessment of Life-Threatening Emergencies—Revised
IVCDI	Inferior vena cava distensibility index
PVR	Pulmonary vascular resistance
PDA	Patent ductus arteriosus
RV	Right ventricle
LV	Left ventricle
PH	Pulmonary hypertension
RVOT	Right ventricular outflow tract
LVOT VTI	Left ventricular outflow tract velocity–time integral
IVC	Inferior vena cava
IVCCI	Inferior vena cava collapsibility index
TAPSE	Tricuspid annular plane systolic excursion
TR	Tricuspid regurgitation
